# Postural Control in Lowlanders With COPD Traveling to 3100 m: Data From a Randomized Trial Evaluating the Effect of Preventive Dexamethasone Treatment

**DOI:** 10.3389/fphys.2018.00752

**Published:** 2018-06-22

**Authors:** Lara Muralt, Michael Furian, Mona Lichtblau, Sayaka S. Aeschbacher, Ross A. Clark, Bermet Estebesova, Ulan Sheraliev, Nuriddin Marazhapov, Batyr Osmonov, Maya Bisang, Stefanie Ulrich, Tsogyal D. Latshang, Silvia Ulrich, Talant M. Sooronbaev, Konrad E. Bloch

**Affiliations:** ^1^Department of Respiratory Medicine, University Hospital Zurich, Zurich, Switzerland; ^2^Kyrgyz-Swiss High Altitude Clinic and Medical Research Center, Tuja-Ashu, Kyrgyzstan; ^3^School of Health and Sports Science, University of the Sunshine Coast, Sunshine Coast, QLD, Australia; ^4^Department of Respiratory Medicine, National Center for Cardiology and Internal Medicine, Bishkek, Kyrgyzstan

**Keywords:** chronic obstructive pulmonary disease, altitude, hypoxia, postural control, dexamethasone, acute mountain sickness

## Abstract

**Objective:** To evaluate the effects of acute exposure to high altitude and preventive dexamethasone treatment on postural control in patients with chronic obstructive pulmonary disease (COPD).

**Methods:** In this randomized, double-blind parallel-group trial, 104 lowlanders with COPD GOLD 1-2 age 20–75 years, living near Bishkek (760 m), were randomized to receive either dexamethasone (2 × 4 mg/day p.o.) or placebo on the day before ascent and during a 2-day sojourn at Tuja-Ashu high altitude clinic (3100 m), Kyrgyzstan. Postural control was assessed with a Wii Balance Board^TM^ at 760 m and 1 day after arrival at 3100 m. Patients were instructed to stand immobile on both legs with eyes open during five tests of 30 s each, while the center of pressure path length (PL) was measured.

**Results:** With ascent from 760 to 3100 m the PL increased in the placebo group from median (quartiles) 29.2 (25.8; 38.2) to 31.5 (27.3; 39.3) cm (*P* < 0.05); in the dexamethasone group the corresponding increase from 28.8 (22.8; 34.5) to 29.9 (25.2; 37.0) cm was not significant (*P* = 0.10). The mean difference (95% CI) between dexamethasone and placebo groups in altitude-induced changes (treatment effect) was -0.3 (-3.2 to 2.5) cm, (*P* = 0.41). Multivariable regression analysis confirmed a significant increase in PL with higher altitude (coefficient 1.6, 95% CI 0.2 to 3.1, *P* = 0.031) but no effect of dexamethasone was shown (coefficient -0.2, 95% CI -0.4 to 3.6, *P* = 0.925), even when controlled for several potential confounders. PL changes were related more to antero-posterior than lateral sway. Twenty-two of 104 patients had an altitude-related increase in the antero-posterior sway velocity of >25%, what has been associated with an increased risk of falls in previous studies.

**Conclusion:** Lowlanders with COPD travelling from 760 to 3100 m revealed postural instability 24 h after arriving at high altitude, and this was not prevented by dexamethasone.

**Trial Registration:** clinicaltrials.gov Identifier: NCT02450968.

## Introduction

Today many settlements worldwide are located at high altitudes (above 2500 m), with regular working places even above 3000 m. Moreover, mountain tourism and air travel are increasingly popular. Therefore, a large number of people, among them also patients with respiratory conditions, are exposed to hypobaric hypoxia. There are concerns that these patients may suffer from altitude-related adverse health effects including impaired postural control (PC), which may lead to falls or impaired performance during various tasks with consecutive accidents. In healthy individuals we have previously observed an impairment in PC already at altitudes of 1630 and 2590 m (Stadelmann et al., 2015). Other studies in healthy volunteers at a higher altitude (Mount Rosa, 4559 m) and in hypobaric chambers (Holness et al., 1982; Cymerman et al., 2001) have also demonstrated worsening of PC. The underlying mechanisms are poorly understood but it is presumed, that hypoxia affects different sensory functions (visual, somatosensory and vestibular) as well as the central nervous system that controls posture-regulating muscles especially in the lower limbs and trunk within a few minutes of exposure to altitudes of 2438 m (8000 ft) or higher (Wagner et al., 2011; Chiba et al., 2016). During sojourns of more than a few hours at high altitude PC may additionally be disturbed by acute mountain sickness (AMS), which causes headache, ataxia, weakness, dizziness, and decrements of alertness.

Chronic obstructive pulmonary disease (COPD) is associated with chronic inflammation and obstruction of the airways, parenchymal destruction of the lung with impaired gas exchange that promotes hypoxemia and increased pulmonary artery pressure (Vogelmeier et al., 2018). Given the high prevalence of COPD, it is expected that many affected patients are undertaking high altitude or air travel thereby suffering from impaired PC, although this has not been specifically studied. Previous observations suggest that elderly persons and patients with COPD suffer from an impaired PC already at sea level (Muir et al., 2010; Porto et al., 2015). Due to their pulmonary gas exchange impairment COPD patients may have more severe hypoxemia than healthy individuals at corresponding altitude. Thus, we reasoned that COPD patients may experience pronounced PC impairments during altitude or air travel with potentially grave consequences such as dangerous falls, and that measures to prevent or reduce this risk would be desirable.

The purpose of the current study was therefore twofold: (1) to test the hypothesis that lowlanders with COPD would experience impairments in PC during a stay at 3100 m and (2) that these impairments could be prevented by treatment with dexamethasone, a drug with potent glucocorticoid action. We selected dexamethasone for this study because glucocorticoids are used to treat COPD exacerbations and because it is effective in prevention and treatment of AMS in healthy mountaineers.

## Materials and Methods

### Study Design and Setting

The current study was performed from June to August 2015 within the scope of a randomized, placebo controlled double blind parallel design trial evaluating effectiveness of dexamethasone in prevention of altitude-related adverse health effects (ARAHE) in lowlanders with COPD traveling to and staying for 2 days at the high altitude (3100 m) clinic of Tuja-Ashu, Kyrgyztan (clinicaltrials.gov Identifier: NCT02450968). The effects of altitude and of dexamethasone on AMS and various other clinical and physiologic outcomes have been reported recently (Furian et al., 2018).

### Participants

Men and women, aged 20–75 years, living in the Bishkek area (Kyrgyz Republic, mean altitude 760 m) diagnosed with COPD according to GOLD guidelines, grades 1–2, FEV_1_/FVC < 0.7 and FEV_1_ > 50% predicted were invited to participate. Exclusion criteria were severe COPD with FEV_1_ < 50% predicted, hypoxemia < 92% at 760 m measured by pulse oximetry, COPD exacerbation, reversible airflow obstruction, a history suggesting asthma or other respiratory disease, diabetes, uncontrolled cardiovascular disease (such as systemic arterial hypertension, coronary artery disease, previous stroke), history of obstructive sleep apnea, pneumothorax in the last 2 months, untreated or symptomatic peptic ulcer disease, or glaucoma and other conditions that might have interfered with protocol compliance including current heavy smoking (>20 cigarettes per day). Participants gave written informed consent. The study was approved by the Ethics Committee of the National Center of Cardiology and Internal Medicine, Bishkek, Kyrgyzstan(01-8/405) and was endorsed by the Cantonal Ethics Committee Zurich, Switzerland.

### Interventions

Participants underwent baseline evaluation in Bishkek (760 m). One to three weeks later, they travelled to the Tuja-Ashu high altitude clinic (3100 m) by minibus within 3–5 h and stayed there for 2 days. On the day before ascent and while staying at 3100 m, participants took 4 mg capsules of oral dexamethasone twice daily or identical looking placebo capsules under the supervision of an investigator. During the study, participants continued their regular medication and no other treatment was allowed. For safety reasons, participants with clinically relevant AMS defined by an AMSc score ≥0.7 (see below), severe hypoxemia (SpO_2_ < 75% for > 30 min, or < 70% for > 15 min), or any other condition requiring an intervention according to the decision of an independent physician were treated with supplemental oxygen and other appropriate means.

### Assessments

A medical history and clinical examination were obtained. AMS was assessed by the environmental symptoms cerebral score (AMSc) comprising 11 questions on AMS symptoms each rated from 0 (not at all) to 5 (extreme) (Sampson et al., 1983). The weighted sum of responses ranges from 0 to 5. Scores ≥0.7 are considered to reflect clinically relevant AMS. Pulse oximetry during rest (SpO_2_, Konica-Minolta PULSOX-300i) and spirometry (EasyOne; NDD, Zurich, Switzerland) were performed.

PC was assessed by a Wii Balance Board (WBB, Redmond, WA, United States), 30 cm × 50 cm in size, as previously described (Clark et al., 2010; Stadelmann et al., 2015). Examinations took place at 760 m and on the second day of the stay at 3100 m, within 20–26 h after arrival. The subjects stood on the WBB on both legs with eyes open and feet positioned in a 30° angle, 20 cm apart. They were instructed to focus on a black dot on the wall, 1.5 m in front of them at eye level, to keep their hands beside the body and stand as still as possible during five tests each lasting 30 s and separated by a resting period of 2–3 min. During the tests, the WBB recorded displacements of the center of gravity of the subject by 4 integrated sensors, using the sampling method and filtering technique described in detail previously (Clark et al., 2017). A customized software (Labview 8.5 National Instruments, Austin, TX, United States) was used to calibrate the WBB with standard weights and to compute the center of gravity path length (COPL), and the mean and SD sway amplitude and velocity in the antero-posterior (AP) and medio-lateral (ML) directions (Clark et al., 2010; Holmes et al., 2013).

### Outcomes and Sample Size Estimation

The main outcome was the center of pressure path length (COPL) and additional outcomes were other variables from PC tests and from clinical and physiological examinations including clinically relevant AMS, severe hypoxemia, and other adverse effects that required an intervention as mentioned above. Since the minimal important difference for COPL and other indices of PC has not been established we based the sample size estimation for this study on previous studies in 51 healthy individuals who showed a significant change in indices of PC measured by the Wii balance board at 2590 m (Stadelmann et al., 2015). In addition, a sample size estimation was performed for the primary outcome of the main trial associated with the current study, the cumulative incidence of ARAHE during the stay at 3100 m. According to these calculations, a minimal number of 100 participants including drop-outs were required to detect a 50% reduction in ARAHE by dexamethasone (Furian et al., 2018).

### Randomization and Blinding

Participants were randomized 1:1 to dexamethasone or placebo treatment by a computer algorithm minimizing for differences in sex, age ≤ or > 50 years, FEV_1_ < or ≥ 80% predicted (Pocock and Simon, 1975).

Study drugs were dispensed by an independent pharmacist in sets of capsules labeled with a concealed code. Participants and investigators were blinded to the assigned treatment until the completion of data analysis.

### Data Analysis

The primary data analysis was performed in the per protocol population of participants who had successful evaluations at both altitudes. In addition, an intention to treat analysis of the main outcome, the COPL, was performed including data from all randomized participants with missing data replaced by multiple imputations. A separate analysis restricted to data from patients >40 years of age was also performed excluding occasional younger participants fulfilling spirometric criteria of COPD but may not suffering from the classical form of the disease. To reduce effects of measurement variability, mean results from the five tests at each location are reported. Occasional individual missing data in one of the five tests were replaced by group medians of the corresponding altitude. Since most variables were non-normally distributed, data are summarized by medians and quartiles. Effect sizes were quantified by Cohen’s d (i.e., *d* = the difference between two altitudes divided by the pooled standard); with values of *d* < 0.2 considered small, 0.5 to 0.8, medium, and > 0.8, strong (Cohen, 1977). The effects of altitude and of the drug were evaluated by computing mean differences and 95% confidence intervals (95% CI). Multivariable regression analysis was performed with the COPL or AP sway velocity as dependent variable and altitude or SpO_2_, dexamethasone, body height, age, FEV_1_ (% predicted) and presence of AMS as independent variables. A *p*-value < 0.05 was considered statistically significant.

## Results

**FIGURE 1:**
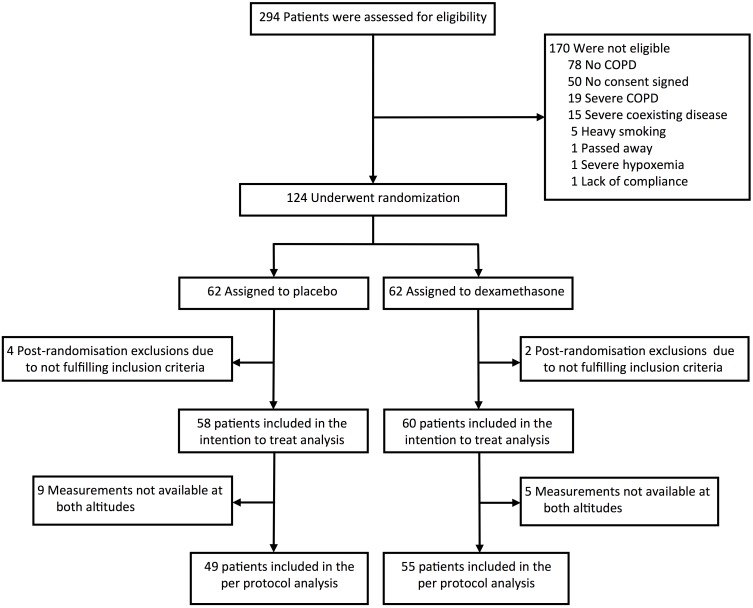
Study flowchart. 124 patients were randomized to dexamethasone respectively placebo. 6 patients were excluded after randomization leaving 118 patients in the intention to treat analysis. In 14 patients, successful balance tests were not available because of altitude-related adverse health effects (2) or incomplete test series (12). 104 patients were included into the per protocol analysis.

A total of 118 patients with COPD fulfilled the inclusion criteria and participated in the study (**Figure 1**). In 2 patients in the dexamethasone group, balance tests at 3100 m could not be performed because of AMS; in 12 additional patients, balance tests were not available at both altitudes for various reasons. Datasets from 104 patients who successfully underwent balance tests at both locations could be included into the per protocol analysis (**Figure 1**). **Table 1** shows the demographic data of the study participants. 70 of the 104 participants (67%) suffered from mild airflow obstruction (GOLD grade 1) and 34 (33%) from moderate airflow obstruction (GOLD grade 2); 97 participants (93%) were >40 years old and 6 participants (7%) were <40 years old (31 to 38 years). 27 of the 118 patients (23%), 13 using dexamethasone, 14 using placebo (*P* = 0.749, Chi-square statistic) suffered from clinically relevant AMS, severe hypoxemia, or other altitude-related adverse health effects.

**Table 1:** Demographic data.

	Placebo	Dexamethasone
*N*	49	55
Sex, male/female	43/6	45/10
Age, years	60 (55; 64)	57 (51; 62)
GOLD grade I/II, *n*	36/13	34/21
FEV_1_, liters	2.7 (2.2; 2.9)	2.5 (1.9; 2.9)
FEV_1_, % predicted	96 (78; 108)	84 (70; 102)
FVC, liters	4.3 (3.7; 4.9)	4.1 (3.3; 4.5)
FVC, % predicted	121 (107; 137)	115 (98; 125)
FEV_1_/FVC	0.65 (0.6; 0.67)	0.63 (0.55; 0.66)
Cigarettes, pack years	25 (2; 38)	20 (0; 34)
BMI, kg/m^2^	24.8 (22.4; 26.8)	25.1 (23.1; 28.0)
Weight, kg	72 (65; 78)	71 (63; 80)
Height, m	1.70 (1.66; 1.75)	1.68 (1.63; 1.73)
SpO_2_ (760 m), %	95 (94; 96)	95 (94; 96)
SpO_2_ (3100 m), %	90 (88; 91)	90 (89; 92)
Medication		
Inhaled bronchodilators, *n* (%)	2 (4%)	1 (2%)
Inhaled corticosteroids, *n* (%)	0 (0%)	0 (0%)
Antihypertensive drugs, *n* (%)	5 (10%)	7 (13%)
Beta-blocker, *n* (%)	3 (6%)	3 (5%)
Aspirin, *n* (%)	5 (10%)	5 (9%)
Antidiabetic drugs, *n* (%)	1 (2%)	0 (0%)

*Values are shown in numbers or median (quartiles); FEV_1_ = Forced expiratory volume in 1 second; FVC = Forced vital capacity; SpO_2_ = arterial oxygen saturation. GOLD = Global Initiative for Chronic Obstructive Lung Disease.*

**FIGURE 2:**
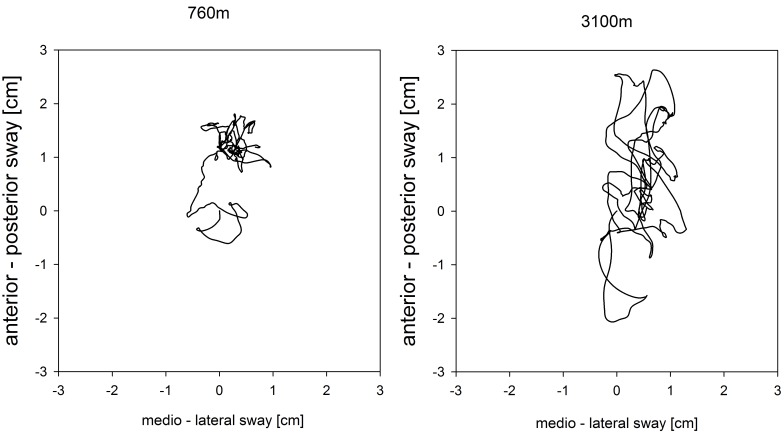
Example of a center of pressure path in an individual COPD patient at 760 m **(left)** and at 3100 m **(right)**. Measurement at 760 m and 3100 m: center of pressure path length (COPL), 21.6 and 39.8 cm; antero-posterior sway velocity, 0.476 and 0.995 cm/s; medio-lateral sway velocity, 0.437 and 0.67 cm/s.

**FIGURE 3:**
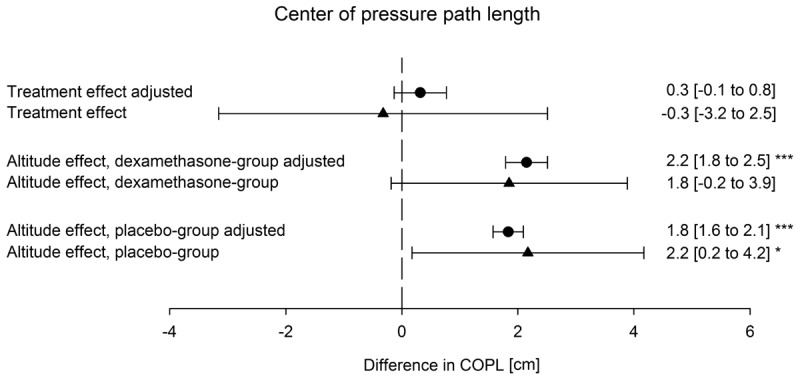
Effect of altitude and of dexamethasone on the center of pressure path length. Altitude effect; difference between the center of pressure path length at 760 m and 3100 m; dexamethasone effect, difference of the altitude effect in the dexamethasone group minus the altitude effect in the placebo group. Mean values and 95% confidence intervals of altitude-induced changes and of differences between dexamethasone and placebo are shown. *^∗^p*-values < 0.05, ^∗∗^*p* < 0.01, ^∗∗∗^*p* < 0.001. Triangles represent raw values, circles represent values adjusted by multiple regression for age, height, weight, forced expiratory volume in 1 second and acute mountain sickness score.

With ascent from 760 to 3100 m the COPL increased significantly in the placebo group from median (quartiles) 29.2 cm (25.8; 38.2) to 31.5 cm (27.3; 39.3) (*P* < 0.05) but in the dexamethasone group the corresponding increase from 28.8 cm (22.8; 34.5) to 29.9 cm (25.2; 37.0) was not significant (*P* = 0.10) (**Table 2**). An example of COPL recordings in a representative individual is shown in **Figure 2**. Mean altitude-induced changes in COPL are shown in **Figure 3**. The mean (95% CI) effect size (Cohen’s d) of the altitude-induced COPL change in the placebo group was *d* = 0.2 (0.02 to 0.39) and in the dexamethasone group 0.17 (-0.02 to 0.37).

**Table 2:** Center of pressure path length at 760 m and 3100 m with placebo and dexamethasone treatment.

	Placebo		Dexamethasone	
		
	760 m	3100 m	760 m	3100 m
Center of pressure path length, cm	29.2 (25.8; 38.2)	31.5^∗^ (27.3; 39.3)	28.8 (22.8; 34.5)	29.9 (25.2; 37.0)
Maximal AP amplitude, cm	2.362 (1.881; 2.806)	2.306 (2.083; 2.679)	2.535 (1.975; 3.113)	2.402 (1.848; 2.914)
AP velocity, cm/s	0.746 (0.645; 0.884)	0.797^∗^ (0.689; 1.030)	0.710 (0.570; 0.907)	0.760^∗^ (0.587; 1.011)
ML velocity, cm/s	0.500 (0.400; 0.665)	0.518 (0.469; 0.702)	0.509 (0.387; 0.598)	0.491 (0.413; 0.641)

*Values are shown in median (quartiles); AP, antero-posterior; ML, medio-lateral; ^∗^p< 0.05 vs. baseline 760 m, paired Wilcoxon rank sum test.*

**FIGURE 4:**
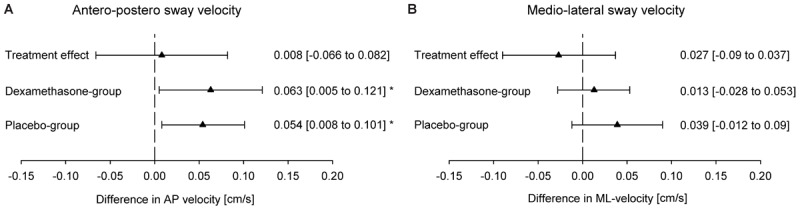
Effect of the altitude and of dexamethasone on sway velocity in the **(A)** antero-posterior (AP) direction and **(B)** medio-lateral (ML) direction. Altitude effect, difference between the center of pressure path length at 760 m and 3100 m; dexamethasone effect, difference of the altitude effect in the dexamethasone group minus the altitude effect in the placebo group. Values are shown as mean (95% CI); ^∗^*p* < 0.05.

Whereas the AP sway velocity increased significantly with ascent to 3100 m in both groups, the medio-lateral sway velocity did not change in any group. Dexamethasone had no significant effect on any index of the PC. The mean difference between dexamethasone and placebo groups in altitude-induced changes in COPL (treatment effect) was -0.3 cm (95% CI -3.2 to 2.5) (*P* = 0.41) (**Figures 3, 4**). The intention to treat analysis of the COPL revealed similar results as the per protocol analysis: the mean altitude-induced change in COPL in the placebo group was 2.1 cm (95% CI 0.2 to 3.9, *P* = 0.028) in the dexamethasone group the corresponding change was 1.8 cm (95% CI 0.01 to 3.7, *P* = 0.049) and the treatment effect was -0.2 cm (95% CI -2.8 to 2.4, *P* = 0.869).

Multivariable regression analysis confirmed a significant increase of 1.6 cm (0.2 to 3.1) [mean (95% CI)] in COPL and of 0.048 cm/s (0.009 to 0.087) in AP sway velocity when ascending form 760 m to 3100 m but no decrease with dexamethasone when controlled for several potential confounders (**Tables 3**, **4**). The mean (95% CI) effect size of the altitude-induced change adjusted for potential confounders listed in **Table 3** was *d* = 0.42 (0.36 to 0.48) in the placebo group and *d* = 0.49 (0.41 to 0.58) in the dexamethasone group. Taking SpO_2_ instead of altitude as the predictor in multivariable regression confirmed that hypoxemia was associated with impaired PC: for each percentage point of reduction in SpO_2_ the COPL was elongated by 0.3 cm (95% CI -0.5 to -0.05, *P* = 0.017) (Supplementary Table S1). A similar negative correlation was found among SpO_2_ and AP sway velocity (Supplementary Table S2).

**Table 3:** Effect of high altitude exposure on the center of pressure path length: multivariable regression.

Dependent variable: Center of pressure path length, cm			
***R*^2^ entire model = 0.180 *P* < 0.001**	**Coefficient**	**95% CI**	***P*-Value**
Altitude (1 = 760 m; 2 = 3100 m)	1.6	0.2 to 3.1	0.031
Drug (1 = Plc; 2 = Dex)	-0.2	-4.0 to 3.6	0.925
Age, years	0.4	0.2 to 0.6	0.001
Sex (1 = men; 2 = women)	1.1	-6.1 to 8.4	0.760
Height, cm	0.5	0.2 to 0.8	0.003
FEV_1_, % pred.	-0.1	-0.2 to 0.0	0.082
AMS (1 = No; 2 = Yes)	3.4	-1.3 to 8.8	0.144
Intercept	-61.7	-123.3 to -0.0	0.050

*Plc, Placebo; Dex, Dexamethasone; FEV_1_, % pred., Forced expiratory volume in 1 second in % of the predicted FEV_1_; AMS, development of acute mountain sickness during altitude exposure assessed by the environmental symptoms score ≥0.7.*

Dexamethasone had no significant effect in the multivariable regression on the COPL, AP- or ML-velocity (**Tables 3**, **4** and Supplementary Tables S1, S2). However, age and body height were associated with increased COPL and AP-velocity.

The presence or absence of AMS during the whole stay at altitude had no significant influence on the COPL. However, the movements in AP-direction, which are more influenced by changes of altitude, were significantly increased in those participants suffering from AMS (Supplementary Tables S2, S3).

In a regression analysis including the ten consecutive performed tests (five tests at 760 m and the five at 3100 m) no learning effect was observed (Supplementary Table S4).

Restricting the regression analysis to the 97 participants > 40 years old revealed similar results as those in **Tables 3**, **4** including all 104 participants, i.e., there was a significant effect of altitude on COPL and AP sway velocity but no significant effect of dexamethasone (Supplementary Tables S5, S6).

**Table 4:** Effect of high altitude exposure on the antero-posterior sway velocity: multivariable regression.

Dependent variable: AP sway velocity, cm/s			
***R*^2^ entire model = 0.180 *P* < 0.001**	**Coefficient**	**[95% CI]**	***P*-Value**
Altitude (1 = 760 m; 2 = 3100 m)	0.048	0.009 to 0.087	0.016
Drug (1 = Plc; 2 = Dex)	0.006	-0.104 to 0.116	0.915
Age, years	0.011	0.004 to 0.018	0.001
Sex (1 = men; 2 = women)	-0.037	-0.231 to 0.157	0.708
Height, cm	0.012	0.003 to 0.020	0.009
FEV_1_, % pred	-0.003	-0.007 to 0.000	0.078
AMS (1 = No; 2 = Yes)	0.099	-0.014 to 0.212	0.085
Intercept	-1.577	-3.385 to 0.230	0.087

*Plc, Placebo; Dex, Dexamethasone; FEV_1_, % pred., Forced expiratory volume in 1 second in % of the predicted FEV_1_; AMS, development of acute mountain sickness during altitude exposure assessed by the environmental symptoms score ≥0.7.*

## Discussion

We studied the effects of acute high altitude exposure (3100 m) and of preventive dexamethasone treatment on postural control (PC) in lowlanders with mild to moderate COPD (GOLD grade 1 – 2). Our randomized, placebo controlled, double-blind trial demonstrates that measures of PC, including AP sway velocity and COPL increased at the higher altitude, consistent with impaired PC during acute exposure to hypobaric hypoxia. The results further revealed that the altitude-induced postural instability was not prevented by treatment with dexamethasone.

The current study is the first evaluating PC in lowlanders with COPD ascending rapidly to high altitude. It is generally assumed that the adverse effects of altitude exposure are related to hypoxia in the nervous system and possibly muscles (Chiba et al., 2016). Consistently, multiple regression analyses taking several independent variables into account (dexamethasone, age, sex, FEV_1_ in % predicted, body height) confirmed an independent negative effect of SpO_2_ on COPL. Previous studies on PC in various simulated and real altitudes have included small numbers of mainly young, healthy volunteers and were conducted in various settings, with different protocols and devices. In the largest study, a randomized cross-over trial, performed in 51 healthy young men staying for 2 days each at 1630 and 2590 m in the Alps, an altitude-related increase in COPL mainly due to AP sway was observed (Stadelmann et al., 2015). Consistently, in the current study, as well as in other previous studies, postural stability was mainly impaired in the AP direction, while ML sway was unchanged at altitude. Other studies using different protocols and tests evaluated effects of much shorter exposures to normobaric hypoxia (minutes to a few hours) at altitude ranging from 2438 m to 5906 m and generally confirmed impairment of PC.

In one study, sensomotoric, visual, and vestibular components of hypoxia-induced impairment of PC were evaluated in combination and separately by varying experimental conditions such as visual inputs (eyes open/closed, sway-referenced visual field), resting and moving platform. The results of these investigations confirmed that static PC (eyes open) and reaction time to unexpected movements of the platform were impaired even during very short exposures of less than 1 h to altitudes equivalent to 2438 m and 3048 m (Wagner et al., 2011).

While most previous studies were performed in young volunteers between 21 and 56 years of age, Drum et al. (2016) evaluated the combined mild normobaric hypoxia (altitude equivalent 2600 m) and exercise (a 40 min treadmill walk) in a group of 37 healthy seniors (mean ± SD age 62 ± 4 years). Balance tests did not reveal an effect of simulated altitude at rest but COPL were larger immediately after exercise at near sea level conditions and even more so after exercise at simulated altitude, suggesting a combined effect of muscular fatigue and hypoxia on PC in these elderly individuals. The current study confirms and extends these observations in a different setting in COPD patients by demonstrating an independent negative effect of altitude and older age on PC even at rest without prior exercise (**Tables 3**, **4**). Since COPD results from long-term cumulative noxious exposures it is most commonly observed in older individuals (>40 years). In the current study 7/104 (6%) participants were between 31 and 38 years old, an age that is not typically compatible with the classical form of COPD. We speculate that these individuals might have suffered from infections and exposure to smoke and indoor air pollution since early childhood. Such “disadvantage factors” may have promoted irreversible airflow obstruction at a relatively young age (Vogelmeier et al., 2018). Excluding data from participants <40 years of age from analysis confirmed a robust effect of altitude on PC in the 97 older COPD patients (>40 years old).

Since regression analysis of data in the current study indicates that altitude as well as age and body height adversely affects PC, it is important to take this into account when assessing PC. Moreover, the study by Drum et al. (2016) suggests that prior exercise may also affect PC, which may be particularly relevant for older individuals desiring to hike in the mountains.

Postural imbalance and ataxia are established diagnostic criteria for severe AMS and high altitude cerebral edema (HACE) (West, 1996; Hackett and Roach, 2004). The AMS questionnaire includes items like “dizziness/lightheadedness” or “my coordination is off” and the heel-to-toe walking as elements related to postural balance (Sampson et al., 1983; Sutton et al., 1992). However, studies on the relation among postural instability at altitude and AMS are controversial. Most studies could not show a relation between AMS and PC (Baumgartner et al., 2002; Cymerman et al., 2001). In the current investigation, multiple regression analysis did not reveal consistent results. Whereas AMS was not a significant predictor of the COPL, it was significantly correlated with the AP-velocity and AP-amplitude. We cannot exclude that the lack of a significant association between COPL and AMS was related to the low prevalence of AMS in our study. Further investigations are required to better define interactions among AMS and PC measured objectively by a Wii balance board and other techniques in order to determine clinically relevant changes in objective indices of PC at high altitude and their relation to altitude-related illnesses including AMS.

To date, data on prevention or treatment of impairment in PC at altitude are scant. Baumgartner and coworkers administered supplemental oxygen to volunteers at 4559 m but failed to detect a beneficial effect on PC although symptoms of AMS were reduced by the intervention (Baumgartner and Bartsch, 2002). These authors therefore suggested that postural ataxia at altitude resulted from different hypoxia-dependent mechanisms than AMS. In the current study we used dexamethasone to evaluate whether it might prevent impairments in PC, AMS, and other altitude-related adverse health effects such as insomnia and cognitive deficiencies in COPD patients. The reason for the lack of a protective effect of dexamethasone on PC remains unexplained. For safety reasons, the study protocol required that all participants suffering from clinically relevant AMS, severe hypoxemia or other adverse health effects were treated with supplemental oxygen and other appropriate means. These predefined precautions may have introduced a “survivor effect” preventing the observation of more severe PC impairments in the most susceptible persons and, thus, obscuring a significant effect of dexamethasone in the per protocol analysis. However, the intention to treat analysis with replacement of missing data by multiple imputation revealed similar altitude-induced changes in COPL and confirmed absence of a significant effect of dexamethasone thereby not supporting a relevant “survivor effect”.

We cannot exclude that the applied dose of 2 mg × 4 mg dexamethasone/day was insufficient to prevent altitude-induced effects on PC. We selected this dose because it is recommended for AMS prevention in current guidelines and was effective in previous studies (Luks et al., 2014; Zheng et al., 2014). We did not want to use a higher dose of dexamethasone because of potential side effects including hypoglycemia. It is also possible that the only mild impairment of PC we found in the COPD patients reduced the sensitivity of our trial to detect a significant reduction in the altitude-induced impairment. The current results and those from previous studies (Stadelmann et al., 2015) suggest that the Wii balance board is a convenient technique to detect small to moderate altitude-related effect sizes of PC changes under field conditions. However, the accuracy of this simple and inexpensive tool was inferior compared to a laboratory-grade force platform in previous studies (Leach et al., 2014). Therefore, limitations in our measurement technique may have concealed minor effects of dexamethasone on PC even though we tried to minimize instrument-related inaccuracies by repeated calibrations, and by using the same board for all measurements in each individual. Moreover, we computed mean values from five tests in each individual in order to reduce variability in the outcomes.

A limitation of our study is the lack of a healthy, age-matched control group of Kyrgyz individuals, which would have allowed to better assess the independent effect of COPD on PC. The clinical relevance of the severity of PC impairment observed in the current study is uncertain as we were unable to correlate these findings directly to a clinical outcome. Kwok and coworkers have shown that elderly individuals, 60–85 years of age, with a greater (i.e., at the 75th percentile) baseline AP sway velocity measured by a Wii balance board had an approximately twofold greater risk of falls in the following year compared to controls with less (i.e., at the 25th percentile) postural sway at baseline. For comparison, 22 of the COPD patients in this current study had an altitude-related increase in the AP sway velocity of >25% – a change that was associated with a twofold risk of falls in the cited study (Kwok et al., 2015). Comparison of the current to the cited study is hampered by differences in protocol and setting. Nevertheless, it is conceivable that any measurable impairment of PC might increase the risk of falls with potentially grave consequences for mountain travelers.

## CONCLUSION

This is the first study investigating PC in non-acclimatized patients with mild to moderate COPD travelling from their altitude of residence of around 760 m to a high altitude of 3100 m. The main findings were an impairment of PC at altitude as demonstrated by an increase in COPL and AP sway velocity. Dexamethasone in a dose of 2 mg × 4 mg per day did not prevent this impairment.

## Author Contributions

LM contributed to study design, data collection, analysis, and drafting the manuscript. MF, ML, SA, BE, US, NM, BO, MB, StU, TL, SiU, and TS contributed to study design, data collection, and critically revising the manuscript. RC contributed to analysis of data. KB contributed to study design, data analysis, and revision of the manuscript.

## Conflict of Interest Statement

The authors declare that the research was conducted in the absence of any commercial or financial relationships that could be construed as a potential conflict of interest.
