# Supplementary material for: Postural Control in Lowlanders With COPD Traveling to 3100 m: Data From a Randomized Trial Evaluating the Effect of Preventive Dexamethasone Treatment
Source: Front Physiol. 2018 Jun 22;9:752. doi: 10.3389/fphys.2018.00752 (PMC6024910; doi:10.3389/fphys.2018.00752)
Supplement: Supplementary file 1 [file Table_1.docx]

**SUPPLEMENT**

**Postural control in lowlanders with COPD travelling to 3100 m; data from a randomized trial evaluating the effect of preventive dexamethasone treatment**

Lara Muralt^1,2^, Michael Furian^1,2^, Mona Lichtblau^1,2^, Sayaka S. Aeschbacher^1,2^, R.A. Clark^4^, Bermet Estebesova^2,3^, Ulan Sheraliev^2, 3^, Nuriddin Marazhapov^2, 3^, Batyr Osmonov^2, 3^, Maya Bisang^1,2^, Stefanie Ulrich^1,2^, Tsogyal D. Latshang^1,2^, Silvia Ulrich^1,2^, Talant M. Sooronbaev^2,3^, Konrad E. Bloch^1,2,3^

^1^ Department of Respiratory Medicine, University Hospital Zurich, Zurich, Switzerland

^2^ Kyrgyz-Swiss High Altitude Clinic and Medical Research Center, Tuja-Ashu, Kyrgyz Republic

^3^ Department of Respiratory Medicine, National Center for Cardiology and Internal Medicine, Bishkek, Kyrgyz Republic

^4^ School of Health and Sports Science, University of the Sunshine Coast, Australia

**Correspondence:**

Konrad E. Bloch

University Hospital Zurich

Dept. of Respiratory Medicine

Ramistrasse 100, CH-8091 Zurich

Switzerland

[konrad.bloch@usz.ch](mailto:konrad.bloch@usz.ch)

| **sTable 1**. **Effect of arterial oxygen saturation (SpO_2_) on the center of pressure path length: multivariable regression** | | | |
| --- | --- | --- | --- |
| **Dependent variable:** **Center of pressure path length, cm** | | | |
| R^2^ entire model= 0.196  P < 0.001 | **Coefficient** | **95% CI** | **P-Value** |
| SpO_2_ | -0.3 | -0.5 to -0.05 | 0.017 |
| Drug (1= Plc; 2= Dex) | -0.0 | -3.8 to 3.8 | 0.988 |
| Age, y | 0.4 | 0.2 to 0.6 | 0.001 |
| Sex (1= men; 2= women) | 1.6 | -5.6 to 8.7 | 0.666 |
| Height, cm | 0.5 | 0.2 to 0.8 | 0.002 |
| FEV_1_, % pred | -0.1 | -0.2 to 0.0 | 0.128 |
| AMS (1=No; 2= Yes) | 4.1 | -0.7 to 9.0 | 0.093 |
| Intercept | -34.3 | -93.4 to 24.7 | 0.254 |
| Plc = Placebo; Dex = Dexamethasone;  FEV_1_, % pred. = Forced expiratory volume in 1 second in % of the predicted FEV_1_;  AMS = development of acute mountain sickness during altitude exposure assessed by the environmental symptoms score ≥ 0.7 | | | |

| **sTable 2. Effect of potential influence factors on antero-posterior sway velocity: multivariable regression** | | | |
| --- | --- | --- | --- |
| **Dependent variable:** **antero-posterior sway velocity, cm/s** | | | |
| R^2^ entire model= 0.195  P < 0.001 | **Coefficient** | **[95% CI]** | **P-Value** |
| SpO_2_ | -0.008 | -0.014 to -0.001 | 0.019 |
| Drug (1= Plc; 2= Dex) | 0.010 | -0.100 to 0.120 | 0.859 |
| Age, y | 0.011 | 0.004 to 0.017 | 0.001 |
| Sex (1= men; 2= women) | -0.026 | -0.216 to 0.165 | 0.792 |
| Height, cm | 0.012 | 0.003 to 0.021 | 0.008 |
| FEV_1_, % pred | -0.003 | -0.006 to 0.001 | 0.113 |
| AMS (1=No; 2= Yes) | 0.115 | 0.006 to 0.223 | 0.039 |
| Intercept | -0.845 | -2.506 to 0.816 | 0.319 |
| Plc = Placebo; Dex = Dexamethasone;  FEV_1_, % pred. = Forced expiratory volume in 1 second in % of the predicted FEV_1_;  AMS = development of acute mountain sickness during altitude exposure assessed by the environmental symptoms score ≥ 0.7; SpO_2_ = Arterial oxygen saturation | | | |

| **sTable 3. Effect of potential influence factors on maximal antero-posterior amplitude: multivariable regression** | | | |
| --- | --- | --- | --- |
| **Dependent variable:** **maximal antero-posterior amplitude, cm** | | | |
| R^2^ entire model= 0.198  P < 0.001 | **Coefficient** | **[95% CI]** | **P-Value** |
| Altitude  (1= 760m; 2= 3100m) | -0.146 | -0.267 to -0.025 | 0.018 |
| Drug (1= Plc; 2= Dex) | 0.181 | -0.021 to 0.382 | 0.079 |
| Age, y | 0.010 | -0.000 to 0.019 | 0.057 |
| Sex (1= men; 2= women) | 0.196 | -0.190 to 0.581 | 0.320 |
| Height, cm | 0.037 | 0.022 to 0.052 | 0.000 |
| FEV_1_, % pred | -0.002 | -0.008 to 0.004 | 0.460 |
| AMS (1=No; 2= Yes) | 0.637 | 0.140 to 1.134 | 0.012 |
| Intercept | -4.452 | -7.681 to -1.223 | 0.007 |
| Plc = Placebo; Dex = Dexamethasone;  FEV_1_, % pred. = Forced expiratory volume in 1 second in % of the predicted FEV_1_;  AMS = development of acute mountain sickness during altitude exposure assessed by the environmental symptoms score ≥ 0.7; SpO_2_ = Arterial oxygen saturation | | | |

| **sTable 4. Effect of multiple testing on the center of pressure path length: multivariable regression** | | | |
| --- | --- | --- | --- |
| **Dependent variable:** **Center of pressure path length, cm** | | | |
| R^2^ entire model= 0.196  P < 0.001 | **Coefficient** | **[95% CI]** | **P-Value** |
| Consecutive tests  (1 to 10) | 0.03 | -0.3 to 0.3 | 0.858 |
| Altitude  (1= 760m; 2= 3100m) | 1.6 | -0.3 to 3.5 | 0.108 |
| Drug (1= Plc; 2= Dex) | -0.2 | -3.9 to 3.6 | 0.932 |
| Age, y | 0.4 | 0.2 to 0.6 | 0.001 |
| Sex (1= men; 2= women) | 1.2 | -6.0 to 8.4 | 0.741 |
| Height, cm | 0.5 | 0.2 to 0.7 | 0.002 |
| FEV_1_, % pred | -0.1 | -0.2 to 0.0 | 0.080 |
| AMS (1=No; 2= Yes) | 3.0 | -1.7 to 7.7 | 0.215 |
| Intercept | -61.2 | -122.0 to -0.4 | 0.048 |
| At both 760 m and at 3200 m, 5 consecutive balance tests were performed. The repetition of tests reached no significance. No learning effect is observed.  Plc = Placebo; Dex = Dexamethasone;  FEV_1_, % pred. = Forced expiratory volume in 1 second in % of the predicted FEV_1_;  AMS = development of acute mountain sickness during altitude exposure assessed by the environmental symptoms score ≥ 0.7 | | | |

| **sTable 5. Effect of high altitude exposure on the center of pressure path length in patients > 40 years: multivariable regression** | | | |
| --- | --- | --- | --- |
| **Dependent variable:** **Center of pressure path length, cm** | | | |
| R^2^ entire model= 0.180  P < 0.001 | **Coefficient** | **95% CI** | **P-Value** |
| Altitude  (1= 760 m ; 2= 3100 m) | 1.7 | 0.2 to 3.2 | 0.029 |
| Drug (1= Plc ; 2= Dex) | -0.1 | -4.1 to 3.8 | 0.946 |
| Age, y | 0.5 | 0.2 to 0.9 | 0.004 |
| Sex (1= men ; 2= women) | 1.8 | -6.0 to 9.5 | 0.655 |
| Height, cm | 0.5 | 0.1 to 0.8 | 0.005 |
| FEV_1_, % pred. | -0.1 | -0.2 to 0.0 | 0.114 |
| AMS (1=No ; 2=Yes) | 3.1 | -2.3 to 8.5 | 0.259 |
| Intercept | -70.1 | -139.9 to -1.7 | 0.045 |
| Plc = Placebo; Dex = Dexamethasone;  FEV_1_, % pred. = Forced expiratory volume in 1 second in % of the predicted FEV_1_;  AMS = development of acute mountain sickness during altitude exposure assessed by the environmental symptoms score ≥ 0.7 | | | |

| **sTable 6. Effect of high altitude exposure on the antero-posterior sway velocity in patients > 40 years: multivariable regression** | | | | |
| --- | --- | --- | --- | --- |
| **Dependent variable:** **AP sway velocity, cm/s** | | | | |
| R^2^ entire model= 0.180  P < 0.001 | **Coefficient** | **[95% CI]** | **P-Value** |  |
| Altitude  (1= 760 m ; 2= 3100 m) | 0.051 | 0.011 to 0.092 | 0.013 |  |
| Drug (1= Plc ; 2= Dex) | 0.008 | -0.107 to 0.123 | 0.897 |  |
| Age, y | 0.014 | 0.004 to 0.024 | 0.008 |  |
| Sex (1= men ; 2= women) | -0.026 | -0.236 to 0.185 | 0.812 |  |
| Height, cm | 0.012 | 0.003 to 0.022 | 0.012 |  |
| FEV_1_, % pred | -0.003 | -0.007 to 0.001 | 0.107 |  |
| AMS (1=No; 2=Yes) | 0.086 | -0.035 to 0.201 | 0.165 |  |
| Intercept | -1.834 | -3.387 to 0.202 | 0.077 |  |
| Plc = Placebo; Dex = Dexamethasone;  FEV_1_, % pred. = Forced expiratory volume in 1 second in % of the predicted FEV_1_;  AMS = development of acute mountain sickness during altitude exposure assessed by the environmental symptoms score ≥ 0.7 | | | | |
